# Geografische Analysen für evidenzbasierte Public-Health-Interventionen: Das Beispiel Identifikation und Typisierung von Risikoclustern für Masern, Mumps und Röteln

**DOI:** 10.1007/s00103-021-03318-9

**Published:** 2021-04-23

**Authors:** Sebastian Völker, Reinhard Hammerschmidt, Anke Spura

**Affiliations:** 1 Stabsbereich Unternehmensentwicklung, Kassenärztliche Vereinigung Westfalen-Lippe (KVWL), Robert-Schimrigk-Str. 4–6, 44141 Dortmund, Deutschland; 2 Zentrum für Public Health und Versorgungsforschung, Masterstudiengang Public Health, Paracelsus Medical University, Salzburg, Österreich; 3 Referat 2-24 Fortbildung, Qualifizierung, Hochschulkooperation, Bundeszentrale für gesundheitliche Aufklärung (BZgA), Köln, Deutschland

**Keywords:** Räumliche Cluster, MMR-Impfung, Kinder, Transdisziplinare Methodik, Medizinische Geografie, Spatial cluster, MMR vaccination, Children, Transdisciplinary methodology, Health geography

## Abstract

**Hintergrund:**

Idealerweise sollten Gesundheitsangebote und -maßnahmen zur Verbesserung der Impfquoten auf lokale Zielpopulationen, z. B. in räumlichen Clustern, zugeschnitten werden. Bisher wurden räumliche Cluster der Unterimmunisierung jedoch kaum beachtet und auf Basis kleinräumiger Daten typisiert.

**Ziel der Arbeit:**

Anhand des Beispiels der Impfung gegen Masern, Mumps und Röteln (MMR) bei Kindern sollen in der vorliegenden Studie 1. die räumliche Verteilung nicht ausreichender MMR-Impfungen in Westfalen-Lippe kleinräumig identifiziert, 2. spezifische, räumliche Risikocluster mit nicht ausreichendem Impfschutz aufgezeigt und 3. räumlich-nachbarschaftliche Einflussfaktoren der unterschiedlichen Risikocluster als Ansatzpunkte für Public-Health-Interventionen beschrieben werden.

**Material und Methoden:**

Grundlage waren Abrechnungsdaten der Kassenärztlichen Vereinigung Westfalen-Lippe (KVWL). Es wurden Geburtsjahrgangskohorten 2013–2016 von gesetzlich versicherten Kindern gebildet und auf Postleitzahlenebene (*n* = 410) aggregiert. Es wurden statistisch signifikante, räumlich kompakte Cluster und relative Risiken (RR) der Unterimmunisierung identifiziert. Lokale Risikomodelle wurden in binär logistischen Regressionen auf Basis von räumlich-nachbarschaftlichen Variablen geschätzt.

**Ergebnisse und Diskussion:**

Für die Impfquoten „mindestens eine MMR-Impfung“ und „beide MMR-Impfungen“ wurden jeweils 2 signifikante Cluster der Unterimmunisierung identifiziert. Signifikante Risikofaktoren für niedrige Impfquoten umfassten Altersstruktur, sozioökonomische Variablen, Einwohnerdichte, medizinische Versorgung und Werthaltung. Die vorgeschlagene Methodik ist geeignet, räumliche Variationen des Impfverhaltens auf Basis der identifizierten Typologien für gezielte evidenzbasierte Interventionen zu beschreiben.

## Abstract (en)

**Background:**

Ideally, health services and interventions to improve immunization rates should be tailored to local target populations, such as spatial clusters. However, to date, little attention has been paid to spatial clusters of underimmunization and have instead been typified based on small-scale data.

**Aim:**

Using the example of vaccination against measles, mumps, and rubella (MMR) in children, the present study aims to (1) identify the spatial distribution of insufficient MMR vaccination in Westphalia-Lippe on a small scale, (2) identify specific, spatial risk clusters with insufficient vaccination protection, and (3) describe spatial-neighborhood influencing factors of the different risk clusters as starting points for public health interventions.

**Material and methods:**

Account data from the Kassenärztliche Vereinigung Westfalen-Lippe (KVWL) were used as a basis. Birth cohorts 2013–2016 of children with statutory health insurance were formed and aggregated at postcode level (*n* = 410). Statistically significant, spatially compact clusters and relative risks (RRs) of underimmunization were identified. Local risk models were estimated in binary logistic regressions based on spatial-neighborhood variables.

**Results and discussion:**

Two significant clusters of underimmunization were identified for each of the vaccination rates “at least one MMR vaccination” and “both MMR vaccinations.” Significant risk factors for low immunization rates included age structure, socioeconomic variables, population density, medical coverage, and value attitude. The proposed methodology is suitable for describing spatial variations in vaccination behavior based on identified typologies for targeted evidence-based interventions.

## Einleitung

Impfen gehört zu den wirksamsten Maßnahmen des Gesundheitsschutzes [1]. Idealerweise sind für einen bevölkerungsbezogenen Nutzen Gesundheitsangebote und -maßnahmen zur Verbesserung der Impfquoten auf Bevölkerungsgruppen zugeschnitten [1]. Bei evidenzbasierten Krankheitskontroll- und Präventionsprogrammen sind kleinräumige Analysen von Impfraten und kontextuellen Bedingungen nützlich.

Die räumliche Epidemiologie beschreibt und analysiert geografische Variationen von Gesundheitsphänomenen unter Berücksichtigung von demografischen, umweltbezogenen, sozioökonomischen, genetischen und verhaltensbezogenen Risikofaktoren. Der grundlegende Ansatz der räumlichen Epidemiologie geht zurück auf Toblers First Law of Geography, in dem er beschreibt: „everything is related to everything else, but near things are more related than distant things“ [2].

Der Zusammenhang von Durchimpfungsraten und Krankheitsinzidenz wurde wiederholt nachgewiesen [3–5]. Weitergehende Analysen von Impfprozessen beschränken sich aber bislang weitgehend auf deskriptive Choroplethenkartierungen (Flächenkarten als thematische Karten roher und adjustierter Impfraten; [6–8]). Der Einfluss räumlich voneinander abhängiger Gebiete und räumlicher Nachbarschaften wird in mehreren Studien nicht ausreichend beachtet. Beispielsweise bei Feikin et al. [9] in ihrer deskriptiven Analyse des relativen Risikos (RR) von Maserninzidenzraten und Impfverweigerung auf Ebene von Counties in Colorado (USA) und bei Goffrier et al. [6], in Analysen der Compliance zu den Empfehlungen der Ständigen Impfkommission (STIKO) bei der Masernimpfung bei Kindern unter 2 Jahren in Deutschland werden Abhängigkeiten der untersuchten Raumeinheiten untereinander nicht berücksichtigt.

Im Allgemeinen beeinflusst die Nachbarschaft, in der die Menschen leben, das Sozial- und Gesundheitsverhalten tiefgreifend, insbesondere bei Kindern und Jugendlichen [10]. Kleinräumige, auf Nachbarschaften fokussierte Disparitäten des Impfverhaltens sind für die evidenzbasierte Entwicklung von Präventionsprogrammen entscheidend. Die Identifikation dieser Disparitäten mit einfachen, breit anwendbaren evidenzbasierten Methoden ist wichtig, um mögliche Grenzen von Impfprogrammen zu identifizieren, die durch die routinemäßige Überwachung der Durchimpfungsrate nicht erkannt werden können. Die Möglichkeit, räumliche Cluster zu identifizieren, würde Gesundheitsorganisationen und Anbietern von Gesundheitsdienstleistungen bei der Planung und Bewertung von kleinräumigen Maßnahmen zur Vermeidung impfpräventabler Erkrankungen unterstützen. Maßnahmen könnten so lokal fokussiert und auf Zielgruppen bedarfsorientiert konzentriert werden [11, 12]. Der Bedarf ergibt sich aus dem Risiko für ein Ausbruchsgeschehen durch Impflücken, z. B. bei Masern, Mumps und Röteln (MMR) bei Kindern unter 6 Jahren.

Die auslösenden Faktoren für die Variation des Impfverhaltens in verschiedenen Ländern, Gebieten oder Gemeinden können noch nicht vollständig nachvollzogen werden. Im Allgemeinen geht Diekema [13] in einer landesweiten Analyse von Impfraten in den USA von der Hypothese aus, dass geteilte Überzeugungen innerhalb von Gemeinschaften Impfvariationen bedingen. Weitere gemeindebezogene Studien zeigen, dass sozioökonomische Faktoren, Bildung, ethnische Zugehörigkeit und Gesundheitsversorgung eine wichtige Rolle für das Impfverhalten und die Entstehung von Unterimmunisierungsclustern spielen. Völker [14], der Cluster unterimmunisierter älterer Menschen gegen Influenzagrippe in Westfalen-Lippe (WL, Deutschland) ermittelte, und Cadena et al. [15], die MMR-Unterimmunisierungscluster in Minnesota und Washington (USA) analysierten, stellten fest, dass Altersstruktur und Erwerbstätigkeit eine wichtige Rolle in Gebieten einnehmen, die Teil eines Unterimmunisierungsclusters sind. Geremew et al. [16], die eine Studie zur Masernimpfung mit einer sekundären Datenstichprobe von ungefähr 4000 Kindern in Äthiopien durchführten, und Völker [14] sahen in ihren allgemeinen Modellen von Unterimmunisierungsclustern einen signifikanten Einfluss von Bildung und Gesundheitsversorgung (Zugänglichkeit und Häufigkeit der Nutzung). Andere mögliche Faktoren für die Ablehnung von Impfstoffen sind laut Salmon et al. [17] hohes Vertrauen in Fachkräfte der Alternativmedizin sowie geringes Vertrauen in medizinische, gesundheitspolitische oder staatliche Informationsquellen und Organisationen.

Nur in wenigen, neueren Studien werden räumliche Clustermethoden angewandt [12, 14–16, 18–20]. Dabei wurden u. a. keine Wohnortdaten von unterimmunisierten Kindern verwendet, diese mussten z. B. aus der Adresse der besuchten Schule modelliert werden [15, 20]. Andere Studien konnten räumliche Cluster der Unterimmunisierung identifizieren, aber die Cluster erschienen für spezifische Interventionsmaßnahmen zu groß oder es wurde keine Typologie der Unterimmunisierungscluster erstellt [12, 14, 18, 19].

In der räumlich-epidemiologischen Grundkonzeption des vorliegenden Forschungsvorhabens ist die räumliche Verteilung von Impfungen unterschiedlich, was durch begleitende Risikofaktoren erklärt werden soll. Das Forschungsvorhaben adressiert bisherige Forschungsdesiderata und zielt am Beispiel der MMR-Impfung bei Kindern in Westfalen-Lippe (WL) auf die Beantwortung folgender Forschungsfragen:

- Welche räumliche Verteilung kann für Kinder mit nicht ausreichendem MMR-Impfstatus in WL identifiziert werden?
- Welche spezifischen, räumlichen Risikocluster eines statistisch signifikanten, nicht ausreichenden Impfschutzes können für WL identifiziert werden?
- Auf Basis welcher räumlich-nachbarschaftlicher Einflussfaktoren lassen sich die unterschiedlichen Risikocluster für eine Public-Health-Intervention beschreiben?

Die MMR-Impfung ist eine Kombinationsimpfung gegen Masern, Mumps und Röteln. Die STIKO empfiehlt, dass alle Kinder 2 Impfungen i. d. R. ab einem Alter von 11 Monaten erhalten sollen [21]. Die erste Impfung erfolgt zwischen dem vollendeten 11. und 14. Lebensmonat, die zweite Impfung kann frühestens 4 Wochen nach der ersten und soll spätestens vor der Vollendung des 2. Lebensjahres erfolgen.

## Methoden

### Populationsbildung, Datenaufbereitung und deskriptive Statistik

Studiengrundlage bilden pseudonymisierte Abrechnungsdaten nach §295 SBG V der Kassenärztlichen Vereinigung Westfalen-Lippe (KVWL) der Jahre 2013–2018, in der alle Kontakte von Patientinnen und Patienten mit ambulant tätigen Vertragsärztinnen und -ärzten sowie entsprechende Versorgungsleistungen nach Leistungsdatum, behandelnder Ärztin bzw. behandelndem Arzt und Ort der Leistungserbringung dokumentiert werden. Verfügbare Daten umfassen (i) durchgeführte Impfungen, (ii) ärztliche Leistungen, (iii) Wohnortadresse, (iv) Alter und (v) Geschlecht.

Es werden Geburtsjahrgangskohorten von gesetzlich versicherten Kindern der Jahrgänge 2013–2016 gebildet, die während des gesamten Beobachtungszeitraumes in der Region Westfalen-Lippe wohnten. Auf Ebene der fünfstelligen Postleitzahlen (PLZ; *n* = 410) wird nach dem Wohnort der Patientinnen und Patienten aggregiert. PLZ-Gebiete sind, ebenfalls wie bspw. Kreise und kreisfreie Städte, räumlich relativ stabile, geografische Gebietseinheiten. Sie weisen eine durchschnittliche Fläche von 52,4 km^2^ (min. = 1,2 km^2^; max. = 303,0 km^2^) und eine durchschnittliche Einwohnerzahl von 19.926 Personen auf, wobei maximal 48.218 Personen und minimal 5134 Personen in einem PLZ-Gebiet in WL wohnen. Für die spezifischen Clusteranalysen wird für jedes PLZ-Gebiet ein bevölkerungsgewichteter Schwerpunkt berechnet. Zur Berechnung wird ein 100 × 100 m INSPIRE-Rasterdatensatz mit Bevölkerungszahlen der Firma AZ Direct, Stand: August 2018, einbezogen. Die Anzahl der Impfungen (Gebührenordnungspositionen (GOP) 89401B, 89301B, 89113, 89301, 89401A, 89301A) je Patientin bzw. Patient wird über den gesamten Beobachtungszeitraum basierend auf Postleitzahl, Geburtsjahr und Geschlecht aggregiert.

Die Impfquoten werden wie folgt berechnet:

1 $$P=\sum _{i=1}^{n}\left(x_{i}+y_{i}+z_{i}\right),$$

wobei $P$ die Gesamtpopulation, $x_{i}$ Patientinnen und Patienten ohne MMR-Impfung im PLZ-Gebiet, $y_{i}$ Patientinnen und Patienten mit einer Impfung im PLZ-Gebiet und $z_{i}$ Patientinnen und Patienten mit mindestens 2 Impfungen im PLZ-Gebiet darstellen.

2 $$Pvac_1=\frac{\sum _{i=1}^{n}\left(y_{i}+z_{i}\right)}{P}*100,$$

wobei $Pvac_1$ den Anteil der Patientinnen und Patienten mit mindestens einer MMR-Impfung repräsentiert.

3 $$Pvac_2=\frac{\left(\sum _{i=1}^{n}(z_{i}\right)}{P}*100,$$

wobei $Pvac_2$ den Anteil der Patientinnen und Patienten mit beiden MMR-Impfungen repräsentiert.

Die nach Geburtsjahrgang und Geschlecht standardisierten Impfquoten sind auf Choroplethenkarten dargestellt. Für die Clusteranalyse werden die Impfquoten der einzelnen PLZ-Gebiete nach Teilnahmequote an beiden Früherkennungsuntersuchungen (U-Untersuchungen) U6 und U7 analysiert, um Randeffekte zu adressieren. Dies ist vor allem der Fall, wenn Patientinnen und Patienten in Grenzregionen ärztliche Leistungen außerhalb des Zuständigkeitsbereichs der KVWL in Anspruch nehmen. Insgesamt 10 PLZ-Gebiete mit einer Inanspruchnahmequote (mindestens eine U‑Untersuchung; $\bar{x}$ = 89,3 %, SD = 8,5 %) von weniger als 2‑mal die Standardabweichung wurden aus der scanstatistischen Analyse ausgeschlossen.

### Generelle globale und lokale Clustermethoden

Die räumliche Verteilung von Impfquoten wurde zunächst auf ihre Gleichverteilung getestet, um eine signifikante, nicht zufällige Häufung von Fällen in der Studienregion zu identifizieren, indem generelle Clustermethoden angewendet wurden. Für den globalen Clustering-Test wurde der Test auf räumliche Autokorrelation (Morans *I*) mit einer Raumgewichtungsmatrix (entsprechend der Queen Contiguity), die über Schenkel und Eckpunkte angrenzende Polygone einschließt, verwendet. Anschließend wurde anhand des Local Indicator of Spatial Association (LISA; [22]) getestet, ob lokale Cluster nachweisbar waren, die signifikant häufiger unzureichend geimpfte Patientinnen und Patienten als erwartet aufwiesen. Jedoch bot dieser nur einen ersten Hinweis auf die Verortung eines lokalen Clusters, da bei Anwendung von LISA regelhaft falsch-positive Regionen (= Fehlalarm) ausgewiesen werden, v. a. aufgrund des multiplen Testproblems.

### Spezifische Clustermethoden

Kulldorfs Spatial-Scan-Statistik [23] identifiziert statistisch signifikante, räumlich kompakte Cluster von Fällen. Dabei vergleicht sie die beobachtete Verteilung von Fällen mit zufällig verteilten, simulierten Fallzahlen. Diese folgen relevanten Wahrscheinlichkeitsverteilungen, wie z. B. dem Bernoulli-Wahrscheinlichkeitsmodell, das besonders für Fall-Kontroll-Datensätze mit einer hohen Anzahl von Fällen geeignet ist. Hierfür wird ein adjustierbares elliptisches Fenster auf jeden Wohnort der Fälle zentriert. Im vorliegenden Forschungsvorhaben waren dies die bevölkerungsgewichteten Schwerpunkte der PLZ-Gebiete in WL. Diese Fenster bewegten sich systematisch über das gesamte Studiengebiet für den beobachteten Datensatz und die simulierten Datensätze (mittels Monte Carlo Chain und 999 Wiederholungen). Jedes Fenster konnte als mögliches Cluster betrachtet werden und verglich die beobachtete Anzahl von Fällen und Kontrollen mit der simulierten Anzahl von Fällen und Kontrollen. Diese Vierfeldertafel oder Kontingenztabelle konnte für die RR-Berechnung genutzt werden. Jedes mögliche Cluster erhielt somit einen Wert für das RR einer fehlenden Impfung. Eine Likelihood-Ratio-Statistik wurde berechnet, um das wahrscheinlichste Cluster zu identifizieren. Ein Signifikanzwert (*p* Value) wurde für jedes mögliche Cluster berechnet und zugewiesen. Ein oberer und unterer Grenzwert der Anzahl von Patientinnen und Patienten innerhalb eines Clusters wurde auf min. = 1 % der Gesamtpopulation bzw. max. = 10 % der Gesamtpopulation festgelegt.

### Clustertypisierung

Für die soziale Typisierung der signifikanten Risikocluster wurde ein lokales Clusterrisikomodell auf Basis von räumlich-nachbarschaftlichen Variablen für jedes einzelne Risikocluster berechnet. Als abhängige Variable wurde die Zugehörigkeit eines PLZ-Gebietes zu einem Risikocluster als dichotome Variable definiert. Unabhängige Variablen umfassen aggregierte, nachbarschaftsbezogene Variablen, wie sozioökonomischer Status (u. a. Haushaltseinkommen, Arbeitslosenquote, Kaufkraftindex), RKI-Deprivationsindex (berechnet für jedes PLZ-Gebiet nach [24]), Demografie (Altersklassen), Bildung (u. a. Anteil Hochschulreife, Anteil Hochschulabschluss, Anteil ohne beruflichen Abschluss), Haushalte (u. a. durchschnittliche Haushaltsgröße, Anzahl Haushalte), Siedlungsstruktur (u. a. Anteil Siedlungsfläche, Einwohnerdichte), Erreichbarkeit der medizinischen Versorgung (Fahrminuten zur nächsten Arztpraxis, ärztliche Kontakte je Einwohner) und Staatsnähe (Anteil CDU-Wähler bei letzter Landtagswahl 2017). Eine Operationalisierung der unabhängigen Variablen (uV) „Staatsnähe“ kann in der vorliegenden Studie nur eingeschränkt erfolgen. So können durchaus auch Wähler von anderen politischen Parteien als „staatsnah“ gelten. Aufgrund der begrenzten Verfügbarkeit von Daten kann diese uV nur approximiert werden.

**Figure 1:**
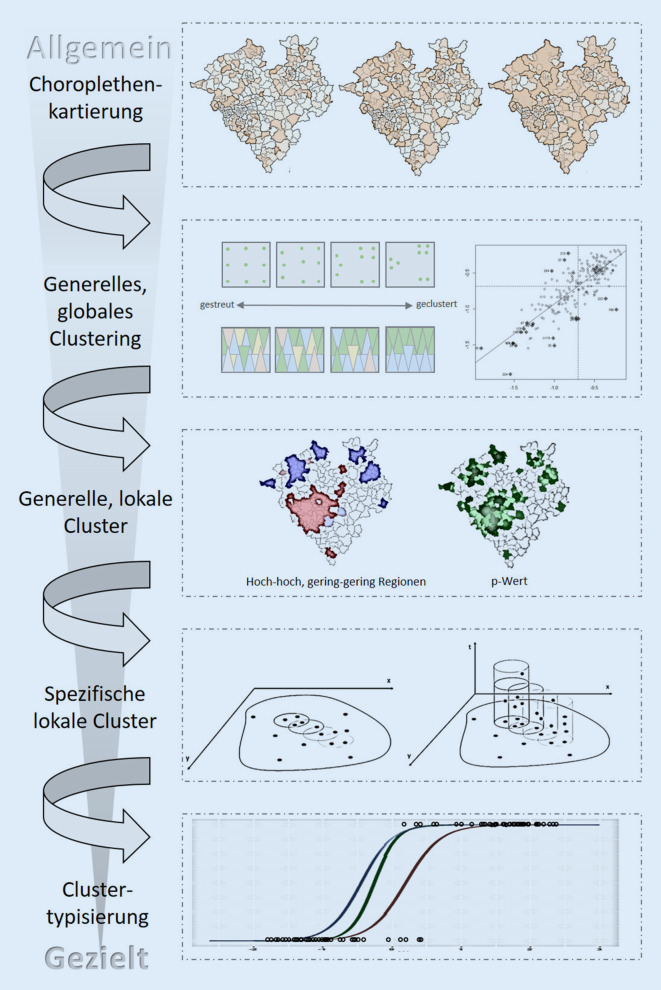

Die Variablen wurden aus dem Data Warehouse der KVWL extrahiert und berechnet oder von der Landesdatenbank Nordrhein-Westfalen (NRW) bzw. der panadress marketing intelligence GmbH erworben und verfügbar gemacht. Die unabhängigen Variablen reflektieren das soziale Umfeld in der lokalen Gemeinschaft ungeimpfter Kinder und führen zu Clustertypologien. Die Analyse wurde als binär logistische Regression auf Basis der auf PLZ-Ebene verfügbaren Variablen der ungeimpften Kinder für jedes identifizierte Cluster durchgeführt, inklusive einer schrittweisen Variablenselektion mittels der Waldstatistik im Ausschlussverfahren („backwards“) und der Bestimmung der Modellgüte die Analyse der Multikollinearität, Devianz (Log-Likelihood), Omnibustest, Histogramm vorhergesagter Wahrscheinlichkeiten, Sensitivität und Nagelkerkes R^2^. Eine Übersicht der Methodik zeigt Abb. 1.

## Ergebnisse

Insgesamt wurden in die Studie 189.737 Kinder der Geburtsjahrgänge 2013–2016 im Alter von 2 bis 5 Jahren eingeschlossen. Dies entsprach einem Anteil von 61,9 % an der gesamten Bevölkerung im Alter von 2 bis 5 Jahren in Westfalen-Lippe. In der Studienpopulation haben im Durchschnitt 94,2 % der Kinder im Studienzeitraum mindestens eine MMR-Impfung, 86,1 % 2 MMR-Impfungen erhalten. Die Kohorte des Geburtsjahrgangs 2016 wies die größte Anzahl an Kindern (*n* = 55.059) auf, die des Geburtsjahrgangs 2013 die geringste (*n* = 40.954). Die Quoten für mindestens eine MMR-Impfung und 2 MMR-Impfungen stiegen vom jüngsten Geburtsjahrgang 2016 bis zum ältesten Geburtsjahrgang 2013 an. Demnach wies die Kohorte 2016 die geringsten (eine Impfung = 92,3 %, 2 Impfungen = 79,6 %) und der Geburtsjahrgang 2013 die höchsten Impfquoten auf (eine Impfung = 95,6 %, 2 Impfungen = 90,3 %).

**Figure 2:**
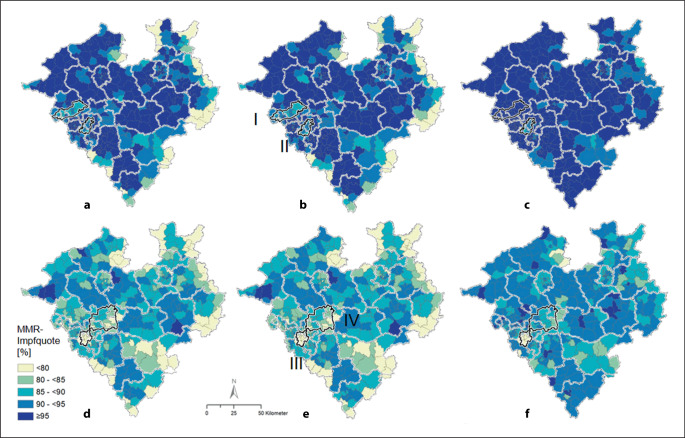

Auf Ebene der Postleitzahlgebiete konnten regionale Unterschiede detektiert werden. Die alters- und geschlechtsstandardisierten MMR-Impfquoten reichten bei mindestens einer Impfung von 35,9 % im Minimum (PLZ-Gebiet 59969, Hallenberg, Hochsauerlandkreis) bis 99,7 % im Maximum (PLZ-Gebiet 58509, Lüdenscheid, Märkischer Kreis) und bei 2 Impfungen von 31,5 % (PLZ-Gebiet 59969, Hallenberg, Hochsauerlandkreis) bis maximal 96,9 % (PLZ-Gebiet 46414, Rhede, Kreis Borken) ohne Adjustierung nach U‑Untersuchungen (Abb. 2b, e). Nach der Adjustierung nach U‑Untersuchungen lagen die Impfquoten für eine Impfung zwischen minimal 87,8 % (PLZ-Gebiet 59909, Bestwig, Hochsauerlandkreis) und maximal 100 % (PLZ-Gebiete 44787, 48629, 59514 und 59555, Stadt Bochum, Metelen, Kreis Steinfurt, Welver, Kreis Soest, und Lippstadt, Kreis Soest) sowie für 2 Impfungen zwischen minimal 65,3 % (PLZ-Gebiet 58455, Witten, Ennepe-Ruhr-Kreis) und maximal 97,8 % (PLZ-Gebiet 33161, Hövelhof, Kreis Paderborn; Abb. 2c, f).

### Räumliche Clusteranalyse

Es konnte eine signifikante, nicht zufällige Häufung von Fällen ohne MMR-Impfung in der Studienregion sowohl für mindestens eine Impfung (Morans *I* = 0,39, *p* < 0,01) als auch für 2 Impfungen nachgewiesen werden (Morans *I* = 0,42, *p* < 0,01). Lokale Cluster wurden ohne Adjustierung bei beiden Clustern an den östlichen und nördlichen Grenzen zu den Bundesländern Niedersachsen und Hessen festgestellt.

**Table 1:** 

Cluster Nr.	Cluster Beschreibung	Anzahl PLZ	Kinder gesamt	Kinder ohne Impfung beobachtet	Kinder ohne Impfung erwartet	Impf-quote innerhalb [%]	Impf-quote außerhalb [%]	Relatives Risiko (RR)	95 %-KI
Mindestens eine MMR-Impfung									
I	Nördliches Ruhrgebiet	19	9692	1067	548	89,0	94,6	2,05	1,92–2,18
II	EN-NO; DO	14	6041	594	341	90,2	94,5	1,78	1,65–1,91
Beide MMR-Impfungen									
III	EN; DO-SW	8	3540	1243	479	64,9	86,9	2,68	2,56–2,80
IV	DO; UN; HAM	24	15271	2653	2066	82,6	86,8	1,32	1,27–1,37

*DO* Dortmund, *DO-SW* Südwest Dortmund, *EN* Ennepe-Ruhr-Kreis, *EN-NO* Ennepe-Ruhr-Kreis Nordost, *HAM* Hamm, *KI* Konfidenzintervall, *PLZ* Postleitzahlen, *UN* Kreis Unna

Für die Impfquoten „mindestens eine MMR-Impfung“ und „beide MMR-Impfungen“ wurden jeweils 2 signifikante Cluster der Unterimmunisierung identifiziert. Diese umfassen das nördliche Ruhrgebiet (*n* = 9692 Kinder gesamt, RR = 2,05 [KI 95 %: 1,92–2,18]) und Dortmund-Ennepe-Ruhr-Kreis (*n* = 6031, RR = 1,78 [KI 95 %: 1,65–1,91]) bzw. Ennepe-Ruhr-Kreis-Dortmund (*n* = 3540, RR = 2,68 [KI 95 %: 2,56–2,80]) und Dortmund-Unna-Hamm (*n* = 15.271, RR = 1,32 [KI 95 % : 1,27–1,37]; Abb. 2b, e sowie Tab. 1).

### Clustertypisierung

Signifikante Risikofaktoren für niedrige Impfquoten umfassten Variablen aus Demografie, Sozioökonomie, Siedlungsstruktur, medizinischer Versorgung und Staatsnähe (Tab. 2). Die identifizierten Cluster wiesen eine unterschiedliche nachbarschaftliche Struktur auf. Bei den demografischen Variablen lag der Anteil von Jugendlichen zwischen 10 und 19 Jahren in allen Clustern unterhalb des Durchschnitts von WL. Der Einwohneranteil der 64- bis 75-Jährigen lag nur in Cluster II unterhalb des Durchschnitts von WL, bei dem Einwohneranteil der über 75-Jährigen waren 2 Cluster oberhalb, 2 Cluster unterhalb des Durchschnitts.

**Table 2:** 

	Mindestens eine MMR-Impfung		Beide MMR-Impfungen		
Variable	Cluster I Nördliches Ruhrgebiet	Cluster II EN-NO; DO	Cluster III EN; DO-SW	Cluster IV DO; UN; HAM	WL Gesamt
Einwohner gesamt	471.624	287.492	177.422	619.147	8.169.999
Anteil 10- bis 19-Jährige (%)	10,1	8,2	9,2	9,6	10,5
Anteil 64- bis 75-Jährige (%)	10,5	9,8	10,8	10,1	9,9
Anteil > 75-Jähriger (%)	11,4	10,4	10,9	10,7	10,8
Sozioökonomischer Deprivationsindex (RKI)	−0,5	−0,2	0,3	−0,5	0,0
Einwohnerdichte (EW/km^2^)	1994	3197	1554	2019	1223
Fahrdistanz kinderärztliche Praxis (min)	4,0	3,3	4,0	2,6	6,6
Inanspruchnahme ärztlicher Leistungen (Tage pro Patient/in und Quartal)	18,8	17,0	18,1	18,2	17,6
Anteil CDU-Wähler Landtagswahl 2017 (%)	26,3	24,8	25,4	28,5	34,1

*CDU* Christlich Demokratische Union Deutschlands, *DO* Dortmund, *DO-SW* Südwest Dortmund, *EN-NO* Ennepe-Ruhr-Kreis Nordost, *HAM* Hamm, *MMR* Masern, Mumps, Röteln, *RKI* Robert Koch-Institut, *UN* Kreis Unna, *WL* Westfalen-Lippe

Der sozioökonomische Deprivationsindex lag in 3 Clustern unterhalb, in einem Cluster oberhalb des Durchschnitts. Bezüglich der Siedlungsstruktur war die Einwohnerdichte in allen Clustern hoch, jedoch in Cluster II mehr als doppelt so hoch wie der Durchschnitt. Die medizinische Versorgung, mit den durchschnittlichen Fahrdistanzen zur nächsten kinderärztlichen Praxis und der Inanspruchnahme ärztlicher Leistungen, unterschied sich nicht signifikant zwischen den Clustern, mit einer Tendenz zu niedrigerer Fahrdistanz. Lediglich Cluster II zeigt eine unterdurchschnittliche ärztliche Leistungsinanspruchnahme. Die die Staatsnähe repräsentierende Variable „Anteil von CDU-Wählern“ lag in allen Clustern unterhalb des Durchschnitts aller Regionen in WL.

In der lokalen Regressionsmodellierung waren in Cluster I (eine Impfung) die Variablen „Anteil der 10- bis 19-jährigen Einwohner“, „Anteil der 64- bis 75-jährigen Einwohner“ und „Inanspruchnahme ärztlicher Leistungen“ (Tage pro Patient/in und Quartal) statistisch signifikant und positiv mit der Zugehörigkeit zum Risikocluster assoziiert. Die Variablen „Sozioökonomischer Deprivationsindex (RKI)“ und „Anteil der CDU-Wähler bei der letzten Landtagswahl“ waren ebenfalls statistisch signifikant, jedoch negativ mit der Zugehörigkeit zum Risikocluster assoziiert.

In Cluster II (eine Impfung) waren die 3 Variablen „Anteil der 10- bis 19-jährigen Einwohner“, „Inanspruchnahme ärztlicher Leistungen“ und „Anteil der CDU-Wähler bei der letzten Landtagswahl“ statistisch signifikant und negativ mit der Zugehörigkeit zum Risikocluster assoziiert.

In Cluster III (2 Impfungen) waren die Variablen „Anteil der 64- bis 75-jährigen Einwohner“ und „Sozioökonomischer Deprivationsindex (RKI)“ statistisch signifikant und positiv assoziiert, während die Variablen „Anteil der 10- bis 19-jährigen Einwohner“, „Anteil der > 75-jährigen Einwohner“, „Einwohnerdichte“ und „Anteil der CDU-Wähler bei der letzten Landtagswahl“ statistisch signifikant und negativ assoziiert waren.

In Cluster IV (2 Impfungen) war die Variable „Anteil der 64- bis 75-jährigen Einwohner“ positiv mit der Zugehörigkeit zum Risikocluster assoziiert, die Variablen „Anteil der > 75-jährigen Einwohner“, „Sozioökonomischer Deprivationsindex (RKI)“ und „Fahrdistanz zur nächsten kinderärztlichen Praxis“ dagegen negativ (Tab. 3). Die Modellgüte war zwischen den Clustern unterschiedlich.

**Table 3:** 

	Mindestens eine MMR-Impfung		Beide MMR-Impfungen	
Variablen (Regressionskoeffizienten $\beta$)	Cluster I Nördliches Ruhrgebiet	Cluster II EN-NO; DO	Cluster III EN; DO-SW	Cluster IV DO; UN; HAM
Anteil 10- bis 19-Jährige	0,5* (0,0–1,1)	−0,7** (−1,1−−0,3)	−0,5* (−1,0–0,0)	n. s.
Anteil 64- bis 75-Jährige	0,6* (0,0–1,3)	n. s.	1,4** (0,4–2,4)	0,8** (0,2–1,5)
Anteil > 75-Jährige	n. s.	n. s.	−1,2** (−2,1–−0,3)	−0,5* (−1,0–−0,0)
Sozioökonomischer Deprivationsindex (RKI)	−1,6* (−2,8–−0,4)	n. s.	1,3* (0,3–2,4)	−1,1** (−1,8–−0,4)
Einwohnerdichte	n. s.	n. s.	−0,9* (−1,9–0,0)	n. s.
Fahrdistanz kinderärztliche Praxis	n. s.	n. s.	n. s.	−0,4** (−0,6–−0,1)
Inanspruchnahme ärztlicher Leistungen	0,02** (0,01–0,04)	−0,02* (−0,01–0,0)	n. s.	n. s.
Anteil CDU-Wähler Landtagswahl 2017	−16,6** (−27,7–−5,5)	−69,4** (−110–−28,1)	−40,5** (−74,9–−6,0)	n. s.
R^2^	0,35	0,51	0,41	0,24

*CDU* Christlich Demokratische Union Deutschlands, *DO* Dortmund, *DO-SW* Südwest Dortmund, *EN-NO* Ennepe-Ruhr-Kreis Nordost, *HAM* Hamm, *MMR* Masern, Mumps, Röteln, *R*^*2*^ Modellgütemaß Nagelkerkes R^2^, *RKI* Robert Koch-Institut, *UN* Kreis Unna, *WL* Westfalen-Lippe, *n. s.* nicht signifikant

## Diskussion

Gesundheitsentscheidungen folgen nur selten einer einzigen Rationalität. Sie sind eingebunden in Werthaltungen, Lebenslagen und Opportunitäten. Bis zur Inanspruchnahme medizinischer Hilfe sind meist Stufen des Hilfesuchverhaltens im sozialen Umfeld, das meist aus medizinischen Laien besteht vorgelagert. Die Inanspruchnahme ist von mehreren Faktoren abhängig, u. a. vom sozioökonomischen Status, Bildung und familiären Merkmalen, wie alleinerziehende Eltern, Kinderzahl oder Alter der Eltern [25]. Das Phänomen des sozialen Einflusses auf individuelle Verhaltensweisen in Nachbarschaften, wie einer Entscheidung für oder gegen eine Impfung, wurde in mehreren Studien beobachtet und beschrieben [26, 27]. Beispielsweise zeigten Rao et al. [28] unterschiedliche Impfquoten je nach Zugang zu Versorgungsangeboten: Nach einer zufälligen Aufteilung von insgesamt 10.091 Master-Studierenden der Universität Harvard von 2002 bis 2006 auf Wohnheime mit und ohne Impfpraxis („flu vaccination clinic“, in denen Studierenden eine Impfung gegen die Influenzagrippe erhalten konnten) zeigte sich, dass die Impfquoten von studentischen Wohnheimbewohnenden mit Impfpraxis höher waren. Wenn 10 % der Bekannten und Freunde eines Individuums in einem Wohnheim mit Impfpraxis wohnten, erhöhte dies die Wahrscheinlichkeit einer Impfung des Individuums um jeweils 1,8 %. Dies hatte kleinräumlich unterschiedliche Impfraten zur Folge. Aus räumlich-zeitlicher Perspektive äußert sich dieses Phänomen zum einen in einer Zunahme von Unterimmunisierungsraten in Gebieten innerhalb räumlicher Cluster als selbstverstärkender Prozess, zum anderen in der verstärkten Zunahme der Unterimmunisierung in Gebieten, die in der Nähe dieser Cluster verortet sind, im Rahmen eines Diffusionsprozesses [19].

Auch wenn Zusammenhänge komplex sind, kann ein ähnliches (Impf‑)Verhalten oft auf geteilte Räume und assoziative Selektion von Individuen zurückgeführt werden [29]. Carpiano [30] entwickelte hierzu ein Konzept auf theoretischer Basis von Bourdieu und Putnam über die Wirkung von nachbarschaftlichen bzw. lokalen sozialen Einflüssen auf Gesundheitsdeterminanten. Hierbei betrachtete er Gesundheitsentscheidungen als sozialen Prozess, anstatt als singuläre Entscheidung.

Die hier identifizierten Einflussvariablen wurden ebenfalls in Studien als signifikante Risikofaktoren für niedrige Impfquoten identifiziert. Die Alterszusammensetzung einer Nachbarschaft bzw. deren Altersstruktur beeinflusst als ein Indikator die zukünftige Impfentscheidung [31–33]. Shaham et al. [32] erkannten signifikante Disparitäten zwischen Impfraten von Patientinnen und Patienten mit unterschiedlichen sozioökonomischen Werten. Die vorliegende Studie bestätigt ebenfalls, dass auch Cluster mit niedrigen Impfquoten sich durch einen hohen sozioökonomischen Status auszeichnen können (Cluster III; [34]). Umgekehrt, in den Clustern I und IV, verringert ein niedriger sozioökonomischer Status die Wahrscheinlichkeit niedriger Impfquoten. Diese Beobachtung deckt sich mit den Ergebnissen von Hegde et al. [11], die über 500.000 Impfstadien von Kindern in Michigan (USA) untersuchten. Kinder in Nachbarschaften mit höherem sozioökonomischen Status hatten eine um rund 1,1 % niedrigere Impfquote bei der Diphtherie-Tetanus-Pertussis-Impfung als Kinder in Nachbarschaften mit niedrigem sozioökonomischen Status.

Die Einwohnerdichte wurde als Faktor ebenfalls bereits in weiteren Studien identifiziert [14]. In der vorliegenden Analyse wurden Risikocluster in hochverdichteten, städtischen Räumen identifiziert. Schon Atkinson und Cheyne [35] wiesen darauf hin, dass aggregierte Daten für urbane Räume häufig Impflücken in kleinräumigeren, aber immer noch stark bevölkerten Gebieten maskieren. In Cluster IV ist eine bessere Erreichbarkeit von Kinderärzten negativ mit der Zugehörigkeit zu einem Risikocluster assoziiert. Dieser Aspekt wurde in mehreren Studien nachgewiesen, in denen die räumliche Erreichbarkeit von Versorgungsangeboten positiv auf die Impfquote wirkt [11, 14]. Die Intensität ärztlicher Inanspruchnahme wurde als negativer Einflussfaktor auf die Impfquote in Cluster I und II identifiziert. Dieser Befund widerspricht jedoch beispielsweise den Ergebnissen von Shaham et al. [32], die eine höhere ärztliche Inanspruchnahme mit einer höheren Impfquote assoziierten. Dies macht die Cluster besonders für eine Public-Health-Intervention interessant, da bereits ein möglicher Zugriff auf die Patientinnen und Patienten seitens des ärztlichen Personals besteht. Schließlich wurde die niedrigere Staatsnähe, mit dem Faktor niedriger Anteil an CDU-Wählern approximiert, als signifikanter Faktor in 3 von 4 Clustern identifiziert. In diesen war eine geringere Staatsnähe mit einer Zugehörigkeit zu einem räumlichen Risikocluster assoziiert, was beispielsweise Feikin et al. [9] bestätigen.

Zusammenfassend sind Ursachen für Impflücken, u. a. bei der Masernimpfung, vielfältig und komplex [31]. Dies findet seinen Ausdruck in den stark unterschiedlichen Clustern, nicht nur in der Deskription nachbarschaftlicher Variablen, sondern auch in der clusterspezifischen Modellierung und Signifikanz von Risikofaktoren. Die Ergebnisse zeigen, dass clusterspezifische Maßnahmen und Lösungen zur Verbesserung der Impfquoten notwendig sind. Für zielgenaue Public-Health-Interventionen liefern die Typisierungen der einzelnen Cluster nicht nur einen klaren Fokus auf vordringliche Interventionsräume, sondern auch die evidenzbasierte Basis für zielgenaue Maßnahmen. Beispielsweise befinden sich in Cluster I Nachbarschaften mit niedrigerem sozioökonomischen Status, in Cluster III Nachbarschaften mit hohem sozioökonomischen Status, die jeweils einer unterschiedlichen Intervention bedürfen. Ein One-size-fits-all-Ansatz wäre nicht zielgruppen- und bedarfsgerecht.

Infektionsepidemiologisch sind Risikocluster besonders gefährlich. Das Potenzial der Krankheitsübertragung ist mit räumlich geclusterten, fehlenden Impfungen größer und erhöht die Gefahr eines zukünftigen Ausbruchs in diesen Gebieten [36]. Geringes Gefahrenbewusstsein ist ein wichtiger Faktor für eine ärztliche Impfaufklärung in Clusternachbarschaften und könnte eine intensivierte individuell-ärztliche Beratung unterstützt von angepassten medialen Aufklärungsinterventionen indizieren.

### Nutzenbewertung für evidenzbasierte Public-Health-Interventionen

Die vorgestellte Beispielanalyse stellt eine räumlich-epidemiologische/medizingeografische Analyse von MMR-Impfraten und Clustern niedriger Impfraten dar und umfasst Theorien und Methoden der deskriptiven und analytischen Epidemiologie. Wenn nun von der Analyse zur Intervention übergegangen wird, um die erkannten Defizite zu reduzieren, kann das Vorhaben im ersten Schritt als retrospektive, analytische Fallkontrollstudie im Querschnittsdesign konzipiert werden. Anschließend wird auf Basis der identifizierten Risikocluster und räumlich-nachbarschaftlichen Variablen eine ökologische Analyse durchgeführt. Nach Atteslander [37] folgt das Vorhaben einem nichtexperimentellen Design. In der räumlichen Epidemiologie existiert kein Instrument zur Sicherung der Qualitätskriterien in Form eines „critical appraisal tool“. Da die Studie eine Fallkontrollstudie beinhaltet, erscheint als geeignetstes Tool die CASP Case Control Study Checklist [38].

Nachteil des Querschnittsdesigns ist, dass die Aussagekraft gegenüber experimentellen Studiendesigns geringer ist. Jedoch ist dieses Studienfeld der räumlichen Epidemiologie bisher weitestgehend unerforscht und wichtige, grundlegende Erkenntnisse zur Konzeption eines experimentellen Designs fehlen. Ziel der Studie ist neben der Methodenerprobung die Entwicklung von Instrumenten für eine evidenzbasierte Gesundheitsplanung und -kommunikation. Die Darstellung einer Assoziation (in Form von Risikoclustern) zwischen Nichtimpfung und Raum ist für die Ableitung spezifischer sektorenübergreifender Maßnahmen aus gesundheitsplanerischer Sicht geeignet. Hierfür ist ein Querschnittsdesign gut geeignet. Die Analyse des Zusammenhangs von räumlich-nachbarschaftlichen (unabhängigen) Variablen und dem Impfverhalten (abhängige Variable) ist für die Ableitung geeigneter Interventionen auf Basis einer evidenzbasierten Entscheidungsfindung aus gesundheitsplanerischer Sicht hinreichend geeignet. PLZ-Gebiete eignen sich in Form einer kleineren Einteilung von Kreisen und kreisfreien Städten für die vorliegende Analyse, jedoch sind diese bspw. für milieufokussierte Studien zu ungenau und nicht detailliert genug. Hier sind andere Raumzuschnitte, wie Marktzellen, Straßenabschnitts- oder Punktdaten besser geeignet. Für gezielte Interventionen zur Verbesserung der Impfquoten sind aus methodischer Sicht eine präzise Datenbasis, anwendungsorientierte Clustergrößen mit einer ausreichenden, jedoch nicht zu großen Anzahl von Patientinnen und Patienten in einem Cluster und eine clusterspezifische Typologie erforderlich.

## Schlussfolgerung und Ausblick

In der vorliegenden Studie wurden die räumliche Analyse und nachbarschaftsbasierte Determinanten des Impfverhaltens zusammengebracht, um Arten von Risikoclustern abzuleiten, bei denen bedarfsgerechte Interventionen zur effizienten Anwendung kommen könnten. Die Identifikation räumlicher Cluster ermöglicht Gesundheitsversorgern und -organisationen Programme zur Reduktion impfpräventabler Erkrankungen räumlich fokussiert zu planen und zu evaluieren. Dabei können verschiedene Zielgruppen nach Interventionsbedarf adressiert werden.

Die verwendeten Methoden sind etabliert und stammen aus anderen akademischen Bereichen wie der Geografie, den Sozialwissenschaften, der Epidemiologie und der Informatik. In dieser Studie haben wir die Methoden in einem innovativen Design kombiniert und empfehlen deren Einsatz als neuartigen Ansatz in der praktischen Gesundheitsfürsorge und Gesundheitskommunikation. Obwohl ein breiter Konsens über zielgruppenorientierte Interventionsprogramme besteht, liegen jedoch nur wenige Studien vor, die räumliche Variation im Impfverhalten aufzeigen. Unsere vorgeschlagene Methodik ist geeignet, räumliche Variationen des Impfverhaltens über verschiedene Typologien für gezielte evidenzbasierte Interventionen zu beschreiben.
